# Rapid situational assessment of people who inject drugs (PWID) in Nairobi and coastal regions of Kenya: a respondent driven sampling survey

**DOI:** 10.1186/s12889-021-11373-9

**Published:** 2021-08-14

**Authors:** Francis O. Oguya, Patrick R. Kenya, Francisca Ongecha, Patrick Mureithi, Helgar Musyoka, Nicholas Muraguri, Ben Mundia, Caleb Angira, Mohammed Shose, Taib A. Basheeb, Abdalla Ahmed Mohamed, John P. Oyore, Otieno G. Ochieng, Gabriel O. Dida, Saade Abdalla, Reychard Abdool

**Affiliations:** 1 Department of Health Systems Management and Public Health, Technical University of Kenya, Nairobi, Kenya; 2 International Centre for Health Interventions Research in Africa (ICHIRA), Nairobi, Kenya; 3 Department of Clinical Medicine, Kenyatta Univerity, Nairobi, Kenya; 4 National AIDS Control Council (NACC), Nairobi, Kenya; 5 National AIDS and STDs Control Programme (NASCOP), Nairobi, Kenya; 6 Nairobi Outreach Services Trust (NOSET), Nairobi, Kenya; 7 OMARI Malindi, Nairobi, Kenya; 8 Reachout Centre Trust, Nairobi, Kenya; 9 Muslim Education Welfare Association (MEWA), Nairobi, Kenya; 10 School of Public Health, Kenyatta University, Nairobi, Kenya; 11 United Nations Office Drugs Crime (UNODC-ROEA), Nairobi, Kenya

**Keywords:** HIV-related behaviors, Injection drug users (PWIDs), Mombasa, Nairobi, Respondent driven sampling, Kenya

## Abstract

**Background:**

A Cross-sectional Rapid Situational Assessment of People Who Inject Drug (PWIDs) applying Respondent Driven sampling techniques (RDS) was used to recruit subjects/participants in a study aimed at assessing HIV prevalence and risk behaviors among injecting drug users in Nairobi and Coastal regions of Kenya. There is paucity of data and information on injecting drug use in sub-Saharan Africa and there is sufficient evidence of existence of the environment for development and growth of injecting drug use. Past studies on PWID and its association to HIV and AIDS that have been conducted in Kenya do not provide sufficient information to support effective planning and comprehensive national response to the HIV and AIDS epidemic.

**Methods:**

A cross-sectional study design was adopted in which a set of initial subjects referred to as ‘seeds’ were first identified from which an expanding chain of referrals were obtained, with subjects from each wave referring subjects of subsequent waves. The seeds were drawn randomly from the population and interviewed to pick the one with the largest network and other unique characteristics. A maximum of twelve seeds were recruited. The second stage involved conducting assessment visits to the sites to identify potential collaborators that included non-governmental organizations (NGOs), drug treatment centres, health facilities, community based organizations (CBO’s) among others. Three NGOs located in the coast region and one in Nairobi region were identified to assist in identifying drug injection locations and potential participants. Key informant interviews (KIIs) and Focus Group Discussions (FGDs) were also conducted using interview guides.

**Results:**

A total of 646 individuals (344 in Nairobi and 302 at the coast) were recruited for the study between January and March 2010. Of these 590 (91%) were male and 56 (9%) were female. Findings showed that most PWIDs initiated injecting drug use between the ages of 20–29 years, with the youngest age of initiation being 11 years and oldest age being 53 years. Most commonly injected drug was heroin (98%), with a small (2%) percentage injecting cocaine. Other non-injecting methods such as smoking or combining these two drugs with other drugs such as cannabis or Rohypnol were also common. Most PWIDs used other substances (cigarettes, alcohol, and cannabis) before initiating injecting drug use. The adjusted national HIV prevalence of PWIDs was 18.3% (19.62% unadjusted) with PWIDs in Nairobi region registering 18.33% (20.58% unadjusted) compared PWIDs for Coastal region indicating 18.27% (18.59% - unadjusted). The gender based HIV prevalence showed that women were more at risk of acquiring HIV (44.51%-adjusted) compared to men (15.97%-adjusted). The age specific HIV prevalence showed that PWIDs who initiated injecting at 11–19 years (44.7% adjusted) were most at risk in Nairobi compared to those who initiated injecting at age 20–24 years (23.2% - adjusted) in the coastal region. While all PWIDs continue to be at risk in the two regions, those from the Western parts of Nairobi, Kenya were at a relatively higher risk given their increased propensity for sharing injecting equipment and solutions.

**Conclusions:**

Compared to the national HIV prevalence of (4.9%), the results show that People Who Inject Drugs (PWIDs) are at particularly high risk of infection in Kenya and there is urgent need for intervention (KenPHIA, 2018). This study also showed clear evidence that 70% of PWIDs are primary school educated, engage in high risk injecting and sexual behaviors comprising sharing of injecting equipment, unprotected heterosexual and homosexual sex. Given that initiation of injecting drug use begins early and peaks after formal school years (20–29 years), prevention programmes should be targeted at primary and secondary school students, college and out of school youth. Further, to protect People who inject drugs (PWIDs) from HIV infection, the country should introduce free Needle Syringe Programs (NSP) with provision of condoms and Methadone Assisted Therapy (MAT) as a substitute for drug use.

## Background

Substance use is increasingly becoming prevalent on the African continent, fueling the spread of HIV infection. Both injection and non-injection substance use constitute the global burden of substance use with Africa having an estimated 28 million substance users [1]. The increasing availability of illicit drugs such as heroin, cocaine and methamphetamine especially in urban areas compound the burden of drug abuse in Africa [2, 3]. Kenya, like other countries in Africa, experiences a high burden of drug use with about 37.1% of the national population reportedly having used a substance in their life time [4].

Globally, about 13 million people inject drugs and about 1.7 million (13.1%) of them are living with HIV [5]. Although benzodiazepines, amphetamine-like substances and opiates are the main classes of injecting drugs, opiates in particular heroin is the most used worldwide by injecting drug users and indeed World Health Organization (WHO) regards drug dependence treatment, particularly opioid dependence treatment, as integral to the scale-up of HIV prevention, treatment, care and support. A study of cohorts of drug users conducted by United Nations Office on Drugs and Crime (UNODC) in 2004 in five major towns found heroin (8.0%) to be the fourth most abused drug after alcohol (36.3%), nicotine (17.5%), and cannabis (9.9%). Cocaine was sixth at 2.2% [6].

With regard to method of drug use, the injecting method was among the popular methods used by a considerable number of drug users. According to Report of the International Narcotics Control Board 2008 [7] and the UNODC World drug report 2007 [8] injecting drug use is likely to increase or emerge in countries where it is not already established. There is cause for concern for regions which lack without adequate resources to deal with the problem. Little is known about injecting drug use in sub-Saharan Africa, but a constellation of risk factors exist for the development of injecting drug use, as has occurred in other regions such as central Asia [9]. First, injecting drug use is already well-established in a number of countries (Mauritius, Nigeria, South Africa, and Tanzania). Secondly, people are experiencing harsh socio-economic conditions and most are exposed to conflict situations and thirdly, the region is increasingly being used for transit of illicit drugs into Europe, all of which are likely to boost the number of injecting drug users. Given that the sub-Saharan Africa is a region with particularly high HIV-1 prevalence and a range of social and biological risk factors, [10] the potential emergence of injecting drug use as an additional route of HIV transmission warrants close and serious attention..

Indeed, risky behavior such as sharing of needles among PWIDs even among those who know that they are positive has been reported in many areas. In 2016, the United Nations International Drug Control Programme (UNDCP), World Drug Report, which is administered by UNODC, indicated that HIV prevalence among PWIDs was high at 68–88% in the sub-Saharan Africa, which compares relatively well with findings from other parts of the world like Myanmar and Spain (66%), Italy (69%), Thailand (80%), implying that the PWIDs may account for a third of new infections in many countries in Sub-Saharan Africa, among them Kenya [11]. The most recent UNODC [1] report, revealed that though regional HIV prevalence rates are high among injecting drugs users in all parts of the world, up to 15.5% them are concentrated in East and Southern Africa. Nearly one-third of new HIV infections, outside Sub-Saharan Africa, are due to PWIDs. Nevertheless, few countries have produced estimates in different time periods to allow for trends to be observed.

Injecting drug use has been documented in Kenya for more than two decades, with evidence showing that the vice contributes significantly to new HIV infections [12]. Initial surveys showed increasing evidence of narcotics use in Mombasa and Nairobi with the practice of poly drug use being more common. A WHO commissioned study conducted in 1999 estimated the number of heroin users, including injecting drug users, at 25,000 and revealed high frequency of multiple sex partners (58%) versus single sex partners (24%). A mapping exercise conducted by four NGOs in 2007 in Nairobi Province and Coast provinces estimated the total number of heroin users to be 12,200 of which 5680 were in Nairobi Province and 6520 in Coast Province [UNODC, 2007]. Crosby et al. [13], demonstrated high risk and prevalence of HIV infections among injecting drug users (IDUs) attending outpatient drug treatment facilities that were only in their infancy in Kenya especially in Nairobi and Mombasa. A limited sample of 120 drug users (101 PWIDs) in Mombasa underwent serological testing for HIV and Hepatitis C. 49.5% tested positive for HIV (all PWIDs) and 70.29% were Hepatitis C positive. The drugs that were injected by this cohort were heroin, cocaine, valium and pethidine.

According to UNAIDS [14], Kenya has among the highest HIV epidemic in the world (alongside Tanzania) with 1.6 million people living with HIV in 2018. In the same year, 25,000 people died from AIDS-related illnesses. Up to 65% of all new infections occurring in nine out of the country’s 47 counties – many of them in the western coast of Kenya [13]. Previous trend showed new HIV infections in major cities of Nairobi and Mombasa increased by more than 50% (from a collective total of 4707 in 2013 to 7145 in 2015). This implies that the HIV prevalence ranges widely from one region to another. For instance Wajir had a prevalence of 0.1% in 2016 compared to the 25.4% reported in Homa Bay in the same period [2, 15].

The core public health message that AIDS is transmissible both through sex and through needle reuse has been taught consistently in developed nations because injection drug use (PWID) is common. However, many AIDS prevention programs in Africa have turned a blind eye on the risk of injection in HIV transmission in their communications with the public, perceiving the practice of injecting drugs as rare. Introducing this information and supporting efficacious infection control in primary health care is vital to protecting patients from HIV as well as other blood borne agents. Given that sharing or use of contaminated syringes and needles is a very efficient means of transmitting HIV, its spread among injecting drug users can be very rapid and even to the general population through sexual contact with people who are not drug users. Whereas limited studies analyzing IDU and its linkages to HIV and AIDS have been conducted in Kenya, they do not provide sufficient updated information necessary to inform an effective action plan for IDU engagement in the national response to HIV and AIDS. Therefore, there is need to conduct a sound scientific contextualised study to obtain reliable data and statistics on the existence of high risk groups such as PWIDs, to understand and document the behaviours and activities that put them at risk of HIV infection and to develop effective prevention strategies and interventions that would address the findings.

Accessing hard-to-reach populations for HIV prevention and research activities has historically been challenging owing to the stigmatization and criminalization of injection drug users, yet these high-risk populations are fundamental in the fight against the spread of HIV. This study therefore adopted the novel Respondent Driven Sampling (RDS) methodology to access hard-to-reach populations through their social networks for purposes of research whose findings can then inform policy [16].

## Methods

### Study sites

The choice of the study sites or study locations is critical to successful recruitment and turn-a-round of respondents into the study. Nairobi is the capital city and is inhabited by Kenyans from all parts of the country while the coastal region comprises of three main cities: Mombasa which happens to be the second largest city after Nairobi, Kilifi and Malindi towns are largely inhabited by the coastal Mijikenda, Swahili people and some few European people who either work or live there. The coastal region is renowned as a popular international tourist destination and has a resident Italian community in Malindi. This study had a total of six study sites located in Nairobi and in the mainland and the Coastal region; there were two sites in Nairobi region (Maisha house/Ngara and Kawangware) and four study sites in the Coast region comprising Mombasa county (Old town/Kisauni, Mariakani/Changamwe and Port Ritz/Nyali/Likoni) and Kilifi county (Mtwapa town and Malindi town). These sites were selected on the basis of existing evidence from previous studies indicating the problem of drug use and especially injecting drug use was rampant in these locations.

### Respondent driven sampling methodology

*Respondent-driven sampling* (RDS), a new form of chain-referral network or snow-ball sampling designed to overcome many of the problems generally attributed to chain-referral sampling such as the choice of initial participants, volunteerism, and masking [17].

These methods are predicated on the recognition that peers are better able than field workers to reach other members of the hidden population. To conduct a respondent-driven sample, one begins by selecting a set of initial seeds that are chosen based on pre-existing contact with the study population. These seeds are paid to be interviewed and from wave 0 of the sample. Sampling begins with a set of initial participants who serve as “seeds,” and expands in waves, where wave 1 consists of participants referred by the seeds, wave 2 consists of participants referred by the first-wave participants, and so each recruitment is a link in the recruitment chain.

Interviews for the recruited injection drug users were conducted in various previously identified and selected research offices in Nairobi and coastal regions. The research office needed to be in a place which was considered safe and accessible to the target population such as existing drop-in-centres, store front or a well-known meeting centre [18]. No matter how the interview was conducted, each seed from wave 0 was supplied with unique recruitment coupons. Subjects were told to give these coupons to other people they knew in the target population. Because each coupon was unique, it can be used to trace the recruitment patterns in the population. When a new member of the target population participates in the study, the recruiter of that person is paid an additional bonus. Thus, subjects are paid to participate and to recruit others.

Hidden populations are often subject to social stigma, criminal prosecution and fear, as a result respondents tend to be hesitant to give information about their colleagues in the population. This phenomenon is referred to as masking [19] and can cause respondents to provide inaccurate information leading to biases in the sample selection process. Normal random sampling procedures are hampered by inherent difficulties in locating respondents for interview. Respondent-driven sampling solves these problems by allowing the subjects to do the recruitment themselves. Participants did not have to divulge any sensitive information on the respondent and the researcher did not have to look for the recruitee. Concerns about introduction of unknown bias into the sampling process due to lack of randomisation resulting from subject based recruitment exists but evidence indicates that respondents recruited randomly from their friends [18].

Critical to the process is the documentation of coupons to record the recruitment patterns linking each respondent to the person who recruited them. There are two additional important steps in the sample selection process: non-duplication and population membership verification. The process must ensure respondents do not participate in the study multiple times in order to earn additional money [20]. This duplication can affect the quality of the data and thus accuracy of the estimates. To ensure quality of the sample data is also critical to verify that sample members are indeed members of the target population. This is done by ensuring that respondents are only counted once and not numerous times [21].

The RDS design is relatively simple and robust and has been used successfully in a number of studies. Reviewers have stated that it is cheaper, quicker, and easier to implement compared to other methods that have been used for sampling hidden populations in evaluations of HIV risk-reduction interventions [22].

### Study design and procedure

**Fig. 1:**
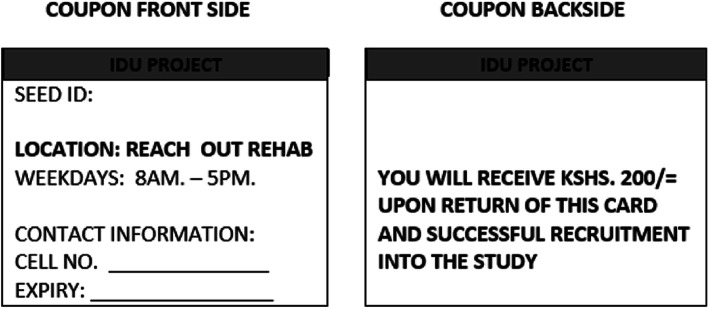
Recruitment coupon (Reach out Rehabilitation centre)

**Fig. 2:**
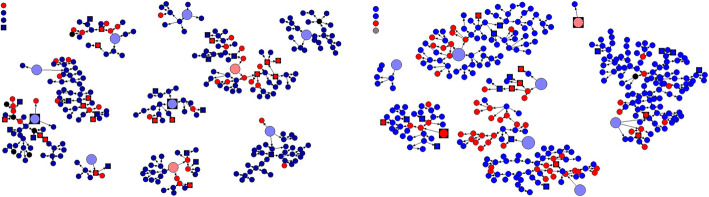
Net Draw Diagram Nairobi and Coast regions. NB: The red large round circles or squares indicate the SEEDs while the small round circles represent males and the small squares represent females. The red colour indicates those who subsequently tested HIV positive and the blue colour are respondents who tested HIV- negative

This was a cross-sectional Rapid Situational Assessment (RSA) study of PWIDs applying the Respondent Driven Sampling (RDS) methodology [18, 19]. The sampling method begun with a set of initial subjects referred to as ‘seeds’ for an expanding chain of referrals, with subjects from each wave referring subjects of subsequent waves. The “seed” subjects were selectively identified from the population and were interviewed to pick those with the largest networks and other unique characteristics such as gender, age group and social status. The RDS method is most suitable when members of the target population know one another and are densely interconnected (chain referral sampling) as is the case with PWIDs. A maximum of twelve seeds were recruited for each of the regions. For this study the quota was set at three and the numbers of waves were also limited to three [19–23]. The quota is the number of persons (coupons) any one participant can recruit (see Fig. 2). These numbers were based on the calculated sample size, projections on potential participants based on the RDS methodology. The purpose of imposing the quota system on recruitment was to reduce respondent duplication and impersonation so as to discourage recruiters from monopolizing recruitment rights. The quota was set at three recruits after the initial interview and each follow-up interview. The quota system was implemented using a coupon system, in which potential recruiters were each given three identification card sized coupons to give to recruits (see Fig. 1). The coupon contained the name of the study, phone number of the researcher (for purpose of enquiries), a serial number that documented the link between the recruiter whom it was given and the recruitee who returns it to the research office as shown on Fig. 1. The Coastal region, where the study was carried out encompassed Mombasa island/city, Kisauni, Kilifi and Malindi. These four sites were located within a diameter 120 km. To ensure faster turnaround time and that respondents were resident within the locality of the site, the seeds and their subsequent recruits were required to be from within their local networks only and given a period of 3 days within which to have distributed and had their recruitment coupons returned. Most respondents were also identifiable by the local community workers who were also working as research assistants in the study. The duration from entry into the survey office and undergoing all procedures to exit ranged from 50 to 75 min.

Apart from the initial waiting time in the survey site, each respondent underwent a screening interview lasting about 5 min, modified WHO Drug Injecting Study II., Version 2b, 2001 questionnaire [24] interview for 30 to 40 min, a voluntary counseling and testing for 20 min, and a tutorial of how to use coupons and receipt of initial incentive for 5 min. Both males and females aged 18 years and older who had been injecting drugs in the last 12 months were included in the study. However, drug users who were ill, experiencing severe withdrawal symptoms or were high on drugs were excluded from the study.

The implementation of the study commenced with visits to Nairobi Outreach Services Trust (NOSET), an NGO that provides outpatient drug treatment services for high-risk injecting and other drug users in Nairobi, to discuss and make collaborative arrangement on implementation of the study. The NOSET representatives were requested to identify appropriate individuals who could be involved in the study as interviewers, coordinators and HIV testing personnel. Several interviews of staff and other individuals were conducted at each centre and seven individuals selected - comprising of five interview staff, two VCT counselors and one coordinator. A 2 day training workshop was conducted in Mombasa and another one in Nairobi for the respective research teams.

### Sample size and data collection tools

Sample size for this study was calculated based on the Fisher et al. [25] formula, assuming a HIV prevalence of 0.66 among PWIDs, which yielded a sample size of 540 individuals [11]. A set of data collection tools were used in this assessment namely: a modified WHO Drug Injecting Study II., Version 2b, 2001 questionnaire, KII guide [26], FGD guide [27], checklist, PWID confirmation screening tool, seed evaluation form and recruitment coupon form some of which were administered at different stages/intervals. The questionnaires were pretested to identify any potential problems in both administration of interviews and logistics and the data collection tools revised appropriately. Data was acquired through a multi-stage approach. The first stage involved the acquisition of information on the magnitude and prevalence of PWIDs, existing policies, the status of service delivery, barriers and opportunities, and gaps in accessing services. This stage comprised of acquisition of secondary data and desk review of publications, documents and reports from key national institutions involved with PWIDs such as NACADA, Ministries of Public Health and Medical Services, national law enforcement (police department), NGOs, donors, NASCOP, NACC, UNAIDS, UNODC, and WHO. The data was collected using a checklist.

The second stage involved conducting assessment visits to the sites to identify potential collaborators that included NGOs, drug treatment centres, health facilities, CBOs, FBOs, Police, Anti-narcotics Police, Hospitals, local Chiefs, Colleges and schools. The purpose of the assessment visits was to determine incidences of drug use and identify presence of injectable drugs and users. Three NGOs in the Coastal region and one in Nairobi region were selected to assist in identifying drug injection locations and potential participants. Focus group discussion and Key informant interviews were conducted with this group using –KII guide. The third stage involved the collection of primary data using a modified version of the WHO drug injecting questionnaire. The modifications to the questionnaire were guided by the analyses of information obtained in the first and second stages. The questionnaire was administered to each respondent by research assistants after which the respondent undertook HIV testing using the serial algorithm for rapid HIV testing according to the National guidelines for HIV testing and Counseling [28].

### Data management and analysis

Data entry was conducted using MS Access database where it was cleaned, verified and double checked to ensure data quality. Descriptive statistics were computed using the Statistical Analyses Software (SAS) version 9.2 [29]. The RDSAT software was used to perform Key of Group and Trait Correspondence, recruitments, transition probabilities, demographically-adjusted Recruitment Matrix, and various options of population estimates [16]. RDSAT software was used to calculate PWID size estimates and to obtain individual data weights which were used to obtain adjusted estimates in subsequent analysis. The netdraw draw diagrams were also drawn using RDSAT software. Bivariate Logistical Regression procedures were carried out using SAS. Association between risky sexual behavior, drug use and HIV status were also performed. Significant relationships between groups were assessed through the χ^2^ statistic, t statistic and odds ratios. Statistical significance was assessed at the conventional probability value of 0.05.

## Results

### Response rate following the RDS strategy – net draws diagrams

A total of ten seeds were recruited in the Coastal region comprising of two women and eight men, while 8 seeds were recruited in Nairobi comprising of two women and six men. In the coast region, four seeds were distributed in Kilifi, Kisauni, three in Mombasa city/Ukunda and three in Malindi/Watamu/Lamu, while in Nairobi eight seeds were recruited and distributed equally between Nairobi east and west regions. Together, these seeds recruited a total of 646 PWIDs over a period of 3 weeks in the Coastal region and 7 weeks in Nairobi region. The convergence factor in the RDSAT software was set at 0.02 and it took four recruitment waves to achieve convergence for all the groups. The minimum network size was 1, the maximum was 60 and the average was 6.7. Each seed resulted in at least 2 waves of recruitment and Homophily in key variables was (0.262 0.3630) indicating that the groups were moderately homogenous. The Net Draws diagrams below (Fig. 2) present the natural structures of client recruitment in the study using the RDS sampling technique, starting with the “seed” to the recruitment of other study participants in subsequent waves.

In general the non-response rate was less than 5% as most participants who were eligible, fulfilled all the requirements of the study and gave informed consent to participate in the study and were enrolled into the study.

### Socio-demographic characteristics of study population

Of the 646 participants recruited in the study, 344 (53.5%) were drawn from Nairobi and 302 (46.7%) from the coast region. Considering the two regions, a total of 178 (27.5%) respondents were recruited in East Nairobi and 166 (25.7%) in Western sections of Nairobi, while in the Coast region, 97 (15.0%) respondents were recruited from Malindi, 92 (14.2%) from Mombasa town, 83 (12.6%) from Kisauni and 30 (4.6%) from Kilifi area.

A majority (590, 91.3%) of the PWIDs were males while 56 (8.7%) were female. Of the female population, 33 (58.9%) were from the Coastal region and 23 (41.1%) from Nairobi region. The youngest PWID in this study was 18 years old and the oldest was 55 years old. Overall adjusted mean and median ages for the whole study population were 31.6 and 31 years, respectively, [IQR: 27–36]. Majority of respondents were aged between 25 and 29 years (29.4%), followed by 30 to 34 years (26.9%), 35–39 years (17.5%), 15 to 19 years (13.9%) and the least comprised 40 to 45 years (12.2%). For Coastal region the women PWID were younger compared to men, their mean age was 27.1 years and their median age was 26 years [IQR: 22–29]. The mean age among coastal men was 32.3 years while the median was 32 years [IQR: 28–37]. As was the case in the Coast region, women PWIDs in Nairobi region were younger compared to men. The mean and median ages for women were 28.2 and 28 years, respectively, [IQR: 24–32]. The mean and median age for men in Nairobi region was 31.8 and 31 years respectively [IQR: 27–36]. Drug use peaked earlier between 25 and 29 years in Nairobi compared to 30 and 34 years in the Coast region.

Over 70% of the respondents were educated to primary school level, 23% to secondary school level, 1.6% had postsecondary education, with 9 respondents being students from local universities, while 3% of the respondents never had any formal education. In relation to marital status, 50% of respondents were single and never married, 30% were either divorced or separated and 9% were married. Of those married, only 9% of the males were in monogamous relationships. With regard to religion, over 80% of PWID in Nairobi were of the Christian faith and 72% of PWID in the Coastal region were of the Islamic faith (Table 1).

**Table 1:** Socio-demographic characteristics (education level, marital status and religion) of respondents in Nairobi and Coast regions

Province	Nairobi County			Mombasa County			All		
	Unadjusted	Women [Adjusted]	Men [Adjusted]	Unadjusted	Women [Adjusted]	Men [Adjusted]	Unadjusted	All Women [Adjusted]	All Men [Adjusted]
**Education Level**									
Never Attended	14 (4.1)	75.9 [75.7–76.0]	3.4 [3.3–3.5]	9 [3.0]	3.4 [3.3–3.5]		23 (3.6)	3.7 [3.7–3.8]	3.7 [3.7–3.8]
Primary	239 (69.1)	24.1 [23.8–24.4]	75.9 [75.9–76.0]	226 [75.6]	75.9 [75.7–76.0]	75.9 [75.7–76.0]	465 (72.1)	73.8 [74.0–74.0]	68.4 [68.4–68.5]
Secondary	87 (25.1)		19.1	60 [20.1]	24.1 [23.8–24.4]	24.1 [23.8–24.4]	147 (22.8)	71.8 [71.7–71.8]	24.8 [24.7–24.9]
College	3.0 (0.9)		1.5 [1.4–1.6]	4.0 [1.3]	1.5 [1.4–1.6]		7.0 (1.1)	1.4 [1.4–1.5]	1.5 [1.4–1.6]
Post secondary	3 (0.9)			.			3 (0.5)	.	
**Marital Status**									
Never Married/ Single	182 (52.7)	39.6 [39.4–39.9]	47.3 [47.2–47.4]	137 [46.1]	27.1 [26.8–27.4]	53.1 [53.1–53,2]	319 (49.7)	25.6 [25.3–25.8]	30.6 [30.6–30.7]
Divorced/ Separated	103 (29.9)	27.1 [26.8–27.4]	30.4.0 [30.3–30.5]	91 [30.6]	23.1 [22.7–23.5]	30.8 [30.7–30.9]	194 (30.2)	25.6 [25.6–25.3]	30.6 [30.6–30.7]
Monogamous	34 (9.9)	15.1 [14.8–15.4]	8.12 [8.0–8.2]	26 [8.8]	0	9.6 [9.5–9.7]	60 (9.3)		50.5 [50.5–50.6]
Married	24 (7.0)	15.2 [14.8–15.4]	11.5 [11.4–11.6]	35 [11.8]	5.2 [5.1–5.3]	5.2 [5.1–5.3]	59 (9.2)	9.3 [9.1–9.6]	0.5 [0.4—0.6]
Widowed	2 (0.6)	2.9[]	1.5 []	5 [1.7]	1.2 [1.1–1,3]	1.2 [1.1–1.3]	7 (1.1)	[1.8]	[1..4]
**Total**	**344 (53.6)**			**302 [46.4]**			**646 (100.0)**		
**Religion**									
Muslim	55 (15.9)	50.8 [50.5–51.1]	23.1 [22.9–24.1]	216 [72.5]	19.3 [19.2–19.4]	74.4 [74.3–74.4]	271 (42.2)	[15.2]	41.7 [41.6–41.7
Roman Catholic	144 (41.7)	24.4 [24.0–24.8]	11.6 [11.8–12.3]	45 [15.1]	18.1 [17.8–18.4]	13.8 [13.6–13.9]	189 (29.4)	[39.4]	28.0 [27.9–28.1]
Protestant	135 (39.1)	24.8 [24.4–25.2	42.7–42.6]	33 [11.1]	14.7 [14,4–15.0]	10.3 [10.2–10.2]	168 (26.1)	[24.2]]	28.2 [28.1–28.3]
Other	11 (3.2)		45.0 [46.0–46.1]	4 [1.3]	18.1 [17.8–18.4]		15 (2.3)	[2.7]	2.1 [2.0–2.2]

**Fig. 3:**
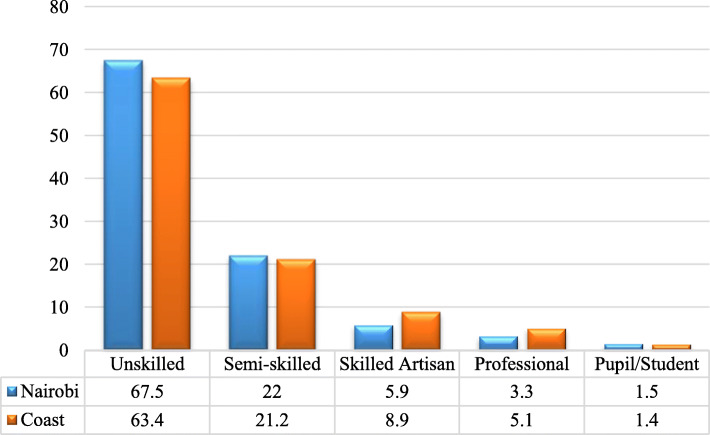
PWID respondents by skills level

Generally, over 60% of the respondents did not have any professional skills, 20% were semi-skilled, 7% were skilled artisans, 4% were professionals while about 2% were pupils/students. Figure 3 below shows skills level per study region.

Respondents’ household incomes in the past 1 month ranged from as low as KShs. 100 to KShs. 50,000. Twenty five percent (25%) of respondents earned up to KShs. 6000, 38% earned between KShs. 6000 and 15,000, 33% earned between KShs. 15,000 and 40,000 in the past month and only 4% of respondents earned over KShs. 40,000. The main source of income was temporary work (55%), while 15% were engaged in self-employment, 14% in criminal activities such as theft and robbery, 4% were in formal employment, 3% were sex workers, 3% were supported by spouses, relatives or friends and 6% had other sources of income (Table 2).

**Table 2:** Respondents’ monthly income levels and source of income

Region	Mombasa		Nairobi		All	
	Number	%	Number	%	Number	%
**Income in Ksh.**						
Up to 6000	67	21.6	75	28.3	142	24.7
6–15,000	125	40.3	94	35.5	219	38.1
16–40,000	103	33.2	86	32.5	189	32.9
45–50,000	14	4.5	9	3.4	23	4.0
**Source of income**						
Temporary work	191	55.4	158	53.6	349	54.5
Self-employed	49	14.2	46	15.6	95	14.8
Theft, robbing or stealing	51	14.8	39	13.2	90	14.1
Regular job with pay	10	2.9	17	5.8	27	4.2
Spouse, partner, relative or friend’s income	11	3.2	8	2.7	19	3.0
Sex work	10	2.9	9	3.1	19	3.0
Other	23	6.7	18	6.1	41	6.4

On average, most (41.3%) respondents reported living with relatives; comprising of either or both parents, grandparents, siblings, aunts and uncles, with the proportion being slightly higher in Nairobi (43.3%) compared to the Coastal region (39.5%). Up to 32.2% of the respondents in the Coast and 25.6% in Nairobi lived alone. Generally, 14.7 and 14.5% of the respondents lived with friends or with their spouses/partners in both regions. Only 0.5% of the respondents did not have a fixed address.

### Sexual orientation of respondents

A large proportion of respondents in Nairobi (91.4%) and Mombasa (95.5%) considered themselves as straight or heterosexual, while 0.7% in Mombasa and 1.6% in Nairobi were bisexual. A total of 3 respondents, 2 in Mombasa and one in Nairobi were lesbians/homosexuals (Table 3).

**Table 3:** Sexual orientation of the respondents

Consider self to be	Region						Total		
	Coast			Nairobi					
	Unadjusted	Adjusted		Unadjusted	Adjusted		Unadjusted	Adjusted	
	N	Females	Males	N	Females	Males	N	Females	Males
Straight or heterosexual	279 (95.2)	88.0 [87.9–88.1]	95.7 [95.7–95.7]	171 (91.4)	100.0 [100–100]	95.5 [95.5–95.5]	450 (93.7)	90.3 [90.2–90.4]	95.6 [95.6–95.6]
Bisexual	2 (0.7)	0	0.8 [0.7–0.9]	3 (1.6)	0	0.8 [0.6–0.9]	5 (1.0)	0	0.8 [0.7–0.9]
Lesbian or homosexual	2 (0.7)	8.9 [8.6–9.3]	0	1 (0.5)	–	–	3 (0.6)	7.2 [6.9–7.5]	0

#### Most preferred drugs and history of drug injection

The most preferred drug of injection among 98% of all the respondents was heroin, with 2% citing cocaine. Heroine and cocaine were also used through other non-injecting methods such as smoking or in combination with other drugs such as Cannabis or Rohypnol. Majority of the respondents reported using other substances like cigarettes, alcohol and cannabis before initiating injecting drug use.

#### Age at initiation

**Fig. 4:**
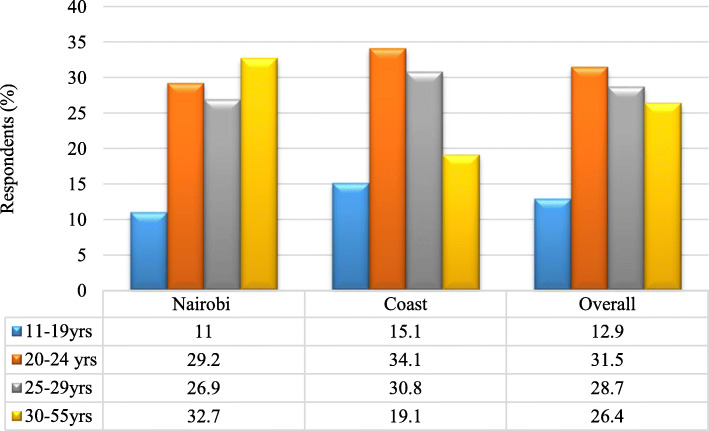
Age at initiation of drug injection

Majority of PWIDs in Coast region initiated injection practice in the 20–24 years age group unlike in Nairobi region where those in 30–55 years age group were the majority (Fig. 4). The mean and median age for commencement of initiation for women was 24.4 years and 20 years respectively [IQR: 20–26). Men initiate injecting at a much later mean and median age of 26.3 and 26 years respectively (IQR: 22–30). Over 30% (33%) of 30–55 years old, 26.9% of 25–29 years old, 29.2% of the 20–24 years old and 11% of the 11–19 years old had initiated drug injection in Nairobi. Whereas for Coast province, majority of injectors were mainly the 20–24 year old age groups at 34.1%, followed by 25–29 year olds at 30.8%, 30–55 year olds at 19.1 and 15.1% for the 30–55 year olds. About two thirds (64.9%) of users in Coast province initiated injecting drug use between ages 20–29 years which is a significant finding compared to 55% in Nairobi. However, the youngest age of initiating injection drug use was 11 years for men and 12 years for women and the oldest was 53 years for men and 49 years for women. Therefore initiation of injection drug use begins early and peaks mainly after formal school years (20–29 years).

#### Person who administered injection at initiation

**Fig. 5:**
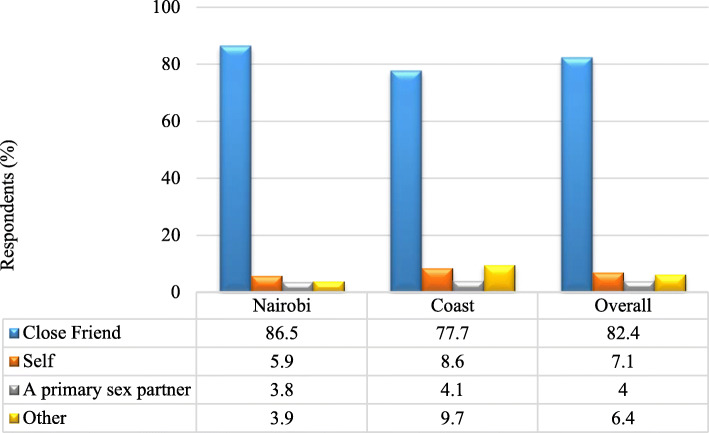
Relationship to person who administered the first injection

All women respondents in the coast region reported getting their first injection administered by a man. However in Nairobi at least 4.3% of the women were injected by fellow women. Figure 5 shows the relationship to person who administered the injection during initiation. In Nairobi 87% compared to 78% in Coast were injected by a close friend, followed by those who self injected at 6% in Nairobi and 9% in Coast. A primary sex partner injected 4.1% and 3.8% in Coast and Nairobi respectively. About 3% in Nairobi compared to 9.7% in Coast were injected by others who included dealer, a friend/acquaintance and a relative.

#### Place of first injection

**Fig. 6:**
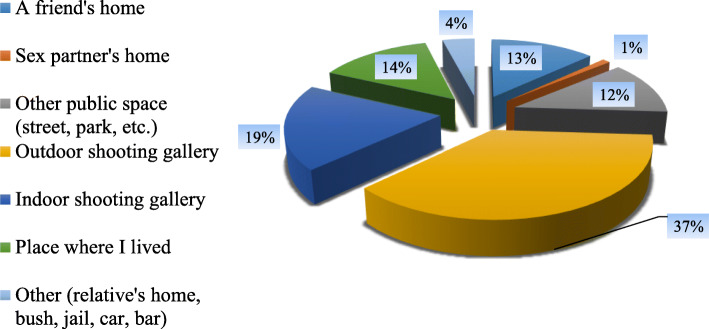
Place of first drug injection

Slightly over a third (37%) of all respondents injected for the very first time in an outdoor shooting gallery, indoor shooting gallery (19%), PWIDs residence (14%), a friend’s home (13%) or other public place (12%) in the two provinces (Fig. 6).

#### Injection practices

Overall, 29.3% of the PWIDs reported sharing needles with about 28.3% of the respondents in Nairobi and 30.4% in the Coast region reported sharing needles. In Nairobi, 82% and 86.5% in Coast injected for the very first time in their current area of residence. Overall, 84.1% injected at their current area of residence (Table 4).

**Table 4:** Prevalence of injecting with used needle and whether injected in current residence

	NAIROBI			COAST			ALL		
	Unadjusted	adjusted		Unadjusted	adjusted		Unadjusted	Adjusted	
	N [%]	Female	Male	N[%]	Female	Male	N[%]	Female	Male
**Injection with used needle**	97 (28.3)	27 [26.7–26.8]	33.5 [33.5–33.6]	90 (30.4)	25.0 [24.7–24.9]	31.0 [31.0–31.2]	187 (29.3)	25.7 [25.6–25.8]	32.5 [32.4–32.6]
**Injection in current area of resident**	283 (82.0)	75.4 [75.3–75.6]	82.5 [82.4–82.5]	256 (86.5)	90.9 [90.9–91.0]	86.1 [86.0–86.1]	539 (84.1)	85.0 [85.0–85.1]	84.1 [84.0–84.1]

#### Reasons for sharing injecting equipment

**Fig. 7:**
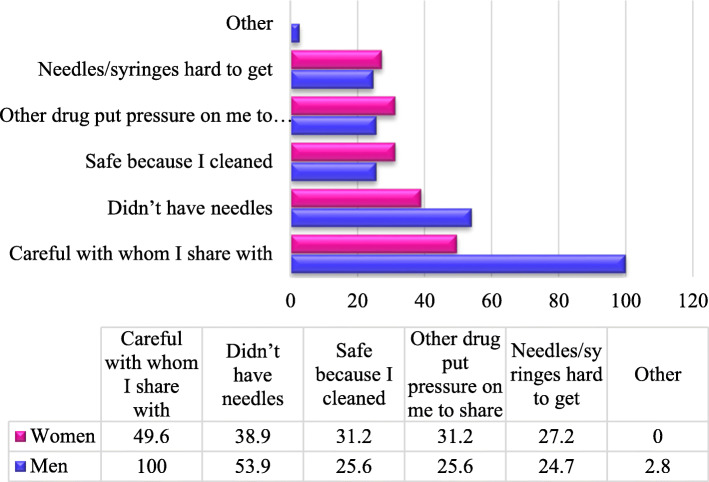
Reasons for sharing needles and syringes

The two most important reasons cited among male and female PWIDs for sharing injecting equipment were being careful on whom they shared the needles and syringes with and lack of their own needles/syringes. Women and men differed on the third reason as men thought it was safe to share since they were cleaning the needles and syringes while women cited pressure from other drug users as their reason for sharing the needles and syringes. Fifty percent (50%) of women cited being careful with whom they shared injecting equipment with, while 100% of men cited the same reason. About 40% of women said that they did not have their own needles and syringes while 100% of men said the same (Fig. 7).

#### Types of reagents used for cleaning injecting equipment

**Fig. 8:**
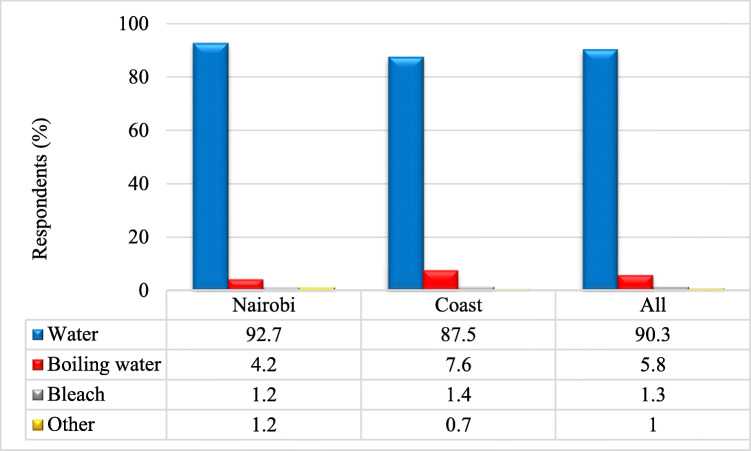
Reagents used for cleaning needles and syringes

Majority used water (92.7% Nairobi, 87.5% Coast and 90.3% overall). The numbers of those who responded were higher than those who shared needles/syringes because they employed more than one method depending on circumstances. Overall, only about 1% used bleach which is the recommended method of cleaning needles/syringes in harm reduction strategies (Fig. 8). Respondents cited the procedure and steps employed in the use of bleach which they consider to be unfriendly as the reason for not using it.

#### Sources of first drug of injection

Overall, 80.4% of the PWIDs bought their own drug, 12.7% got it as a gift, 5.1% gave someone money to buy them the drugs during initiation into injecting drug use. In Nairobi, 80% of respondents bought their own drug, 14% got it as a gift, 4.4% sent someone to buy the drug and only about 1% were dealers or peddlers. None of the PWIDs got drugs as a result of trading for sex. In the Coast region as is Nairobi, majority (81%) bought their own drug, 11.4% received the drug as gifts, 6% sent someone with money to buy the drug, and 0.3% got the drug from a dealer.

### Reasons for initiating injecting drug use

**Fig. 9:**
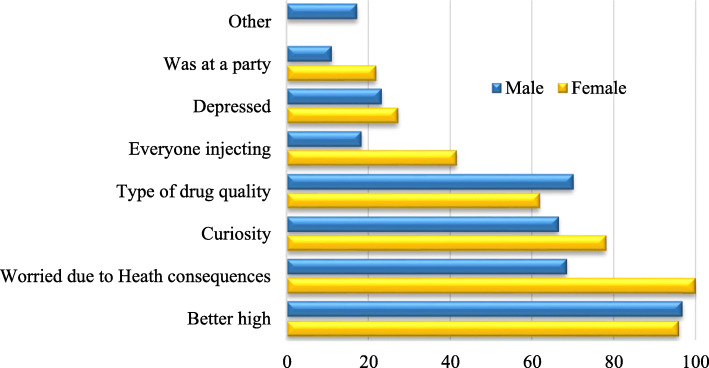
Reasons for initiating injecting drug use

The reasons for initiating injecting drug use varied between the two studied regions. In Nairobi, majority (96.3%) thought injecting would give a better high, and this was mentioned by more than 95% of both gender. Curiosity was mentioned by 78 and 66% of women and men respectively, and drug quality was mentioned by 62% women and 70% men while 100% of women and 69% of men were worried about health consequences. More than 75% said they started injecting because friends and companions were injecting and 67% said that the type or quality of drug available was inadequate for non-injection, 19% injecting because they were worried about the health consequences of other methods of use such as snorting, 64.5% injected out of curiosity, 28.3% due to depression and 18.1% because everyone was doing it (Fig. 9).

In the Coast region, 99.1% thought injecting would give a better high, 86.6% friends/companions were injecting and wanted to try, 84.9% due to pressure from friends/companions and 15% worried about consequences of snorting, 84.6% curiosity, 44.8% due to depression and 25% because everyone else was doing it. The proportion that initiated injection drug use because of curiosity and depression were much higher in Coast than in Nairobi. At initiation, majority thought that they would inject once or twice and then stop, but they continued to inject more regularly as they got addicted to the drugs. Majority have been active injectors in the last 6 months with a few having injection free months.

#### HIV status versus age at initiation of drug injection

**Fig. 10:**
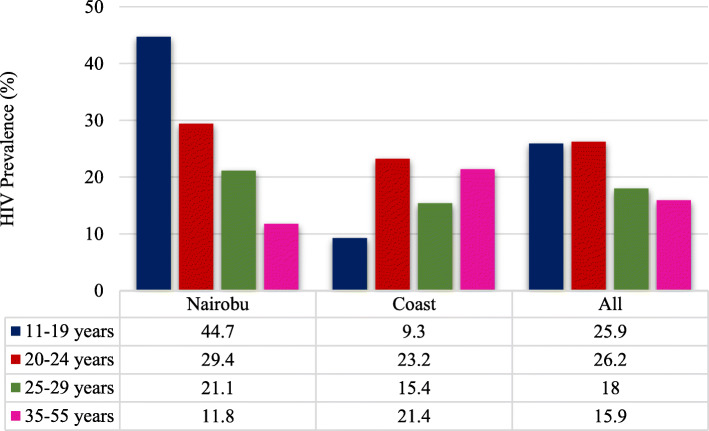
HIV status versus age at initiation of drug injection

HIV testing for each of the study sites is reflected on Table 5. Response was highest in Nairobi compared to Coast where some respondents refused to test or were missing test results for some other reason such as they were incapable of giving consent for testing, or there was a mismatch between the questionnaire and the blood sample, or there was a technical problem in taking the blood sample. In Nairobi 344 respondents were interviewed, 343 were tested, 274 tested HIV negative and 69 tested positive. Out of 302 respondents recruited at the Coast 292 were tested out of which 239 tested negative and 54 tested positive. The overall national HIV prevalence of PWIDs was 18.3% (19.62% unadjusted) with PWIDs in Nairobi region registering higher HIV prevalence of 18.33% (20.58% unadjusted) compared PWIDs for Coastal region indicating 18.27% (18.59% - unadjusted). Despite being fewer in number, women exhibited heightened HIV prevalence of 44.51% (47.27% unadjusted) compared to their male counterparts 15.97% (17.19% unadjusted). Majority of those who initiated injecting drug use at 11–19 years had the highest prevalence of HIV in Nairobi [44.7% adjusted), followed by 20–24 years at 29.4%, 25–29 years at 21.1%. In Coast the highest unadjusted HIV prevalence was seen in those who initiated PWID at 20–24 years (23.2%) which was half the prevalence seen among 11–19 years old initiators in Nairobi. This was followed by 30–55 years at 21.4% (Fig. 10).

**Table 5:** Results of HIV Testing

Study site	Interviewed	Tested	Negative	Positive
East Nairobi	178	177	154	23
West Nairobi	166	166	120	46
**Total Nairobi**	**344**	**343**	**274**	**69**
Malindi, Coast	97	96	75	21
Mombasa town, Coast	92	85	69	16
Kisauni, Coast	83	81	65	16
Kilifi, Coast	30	30	30	1
**Total Coast Region**	**302**	**292**	**239**	**54**
**Overall**	**646**	**635**	**512**	**123**

#### HIV testing among PWIDs

A very high proportion of PWIDs in the study had ever had HIV pre-test counseling. Nairobi recorded 84% versus 77% in Coast with an average of 80% in the overall study. About 84% in Nairobi and 80% in Coast had tested for HIV prior to the study and a high proportion of 93% and 86% in Nairobi and Coast regions respectively had ever received the results of an HIV test, whereas 85% and 78% had received negative HIV test results. A very small proportion of the study population of 11.5% and 15.6% had received HIV positive test results from Nairobi and Coast regions respectively with 13.4% for the overall study population. Of the 27 PWIDs that were aware of having AIDS, not all were on ARV treatment.

#### Future plans and desires of PWIDs

Up to 92% in the Coast region and 95% in Nairobi expected to change their drug consumption in the next 12 months to lesser levels or if possible to quit the habit altogether. Respondents who expected to get employment in the same period of time were 82% from Coast and 90% from Nairobi. Another desire was to have children in which 76 and 73% of the respondents in the Coast and Nairobi regions, respectively, expressed their desire to have children in 12 months’ time. With regard to change in the source of income, slightly more respondents in Nairobi (91%) compared to 82% in the coast region had an expectation of changing their source of income and regarding their health, 94% of respondents in Nairobi and 92% of the respondents in the Coast region expected to have a better health in the next 12 months.

## Discussion

Overall the average HIV prevalence among the PWIDs was comparably high at 19% and most PWIDs appear to be in denial that their injecting drug use behaviour is a major risk factor in HIV infection. Heroine is the main drug of choice for drug injectors and over 90% of PWID are men, initiation of injecting drug use starts early at ages 11–24 years and most were unmarried, have low levels of education and lacked employment skills. This age group also exhibited the highest prevalence of HIV across the two regions. Sharing of needles/syringes and other injecting equipment persists with over a third of all PWIDs confessing this practice.

Results clearly indicated that over 70% of the PWID respondents were educated to primary school level and this is a good indicator for targeting interventions to social networks of in and out-of-school youth with messaging on the dangers, risks and need for avoidance of drug use, curbing the high HIV infection among PWIDs through programs in behavioral change, harm reduction interventions through the Needles Syringe Program (NSP) involving the provision of free syringes, needles and injecting equipment as well as provision of Methadone Assisted Therapy (MAT) or Opioid Substitution Therapy (OST) as a means to overcoming drug dependence.

In the current study, 98% of the PWIDs reported using heroine, while 2% used cocaine, implying that an estimated 9.2 million people across the world take heroin and every year the numbers keep increasing across all continents. The effect that heroin has on the brain chemistry makes it very easy for users to become addicted and very difficult to return to sobriety thus explaining the ever increasing numbers of heroin users. Studies elsewhere have shown that heroin can increase the chance of transition into premature or early regular injection [30], and that the risk of transition to injection among heroin users was higher compared to users of other types of drugs [31, 32]. It has been suggested that heroin, specially injecting type, has a higher degree of dependency compared to other drugs [33]. In addition, heroin is one of the more inexpensive drugs making it easily accessible to a variety of demographics [31]. As such, injecting drug users in Kenya continue to be at high risk like is the case in other parts of the world.

A number of socio-demographic factors influence substance abuse and risk of HIV infection among substance users on the African continent [34]. In the current study, most (91%) PWIDs were men while women accounted for only 9%. The youngest age at initiation was 17 years while the oldest was 55 years age, with the median age at initiation being about 31 years. Studies show that socio-demographic factors such as age, gender, income levels, marital status and even level of education are primary determinants of the health status of drug users [35]. These factors indirectly influence individual drug-use behavior including sharing of needles and soliciting for sex in exchange for drugs or police protection [36, 37]. In the current study for instance, majority of those who initiated injecting drug use at a relatively younger age (11–24 years) had the highest prevalence of HIV in both Nairobi and the coast regions. These current findings are consistent with a study by Baluku et al. [38] that was conducted in two urban centers of Kampala, the capital of Uganda and Mbale Municipality in which the median age at first injection was 19 and a majority reported injecting by the age of 25. These findings however, contrasts with other studies which have reported older ages of first injection drug use for up to 29.87 ± 6.54, for example in Iran [30, 39]. Nevertheless, intervention services in Kenya including prevention of transitioning should target young people with information on relative demerits of commencing drug use and the dangers of peer pressure when associating with drug users.

The current study also established that women are at an increased risk of acquiring HIV compared to their male counterparts and the mean age for initiation of drug injection for women was slightly lower than that of men. Qualitative findings indicated that women were likely to be influenced easily into injecting by their male peers and clients especially if they engaged in sex work. This suggests, in part, that adolescent girls and young women who inject drugs in the study area are likely to transition earlier than their male counterparts. Studies elsewhere have also established that transitioning, like other injecting drug practices have significant gender differences [40–42]. In addition, most of the PWIDs in the age range 11–24, were single and with very low levels of education and over 70% had primary level of education and were unskilled. Studies show that low education among injection drug users is an indicator of a likelihood of needle sharing and non-participation in HIV interventions [43–45]. Consistent with the current study, other studies show evidence that drug-related activity globally has been associated with age, low level of education, familial dysfunction, unemployment, poverty, drug-related violence and gang activity [46, 47].

While different methods of using drugs are associated with social and health harms, injecting - whether intravenous, subcutaneous, or intramuscular, carries the highest risk for multiple types of infections, overdoses, and their complications [48, 49]. Injecting drugs also carries significantly higher risk of HIV and viral hepatitis transmission especially among users who don’t have easy access to sterile injecting equipment and among those who share injecting equipment. Studies indicate that majority of drug users transit from use of non-injection drugs to injection substances or simultaneously use of both substances [50–52]. Furthermore, substance consumption differentially predicts HIV infection. For example, previous studies in Texas, USA (2014) and China (2011) showed that injection substance users have an increased risk of HIV infection in comparison to non-injecting drug users [53, 54].

In the current study, several reasons were put forth for initiating injecting practice among them to achieve a better high, peer pressure from friends and companions, and curiosity among others. Among the motivators for first injection this study, like others elsewhere, underscores the importance of peer influence cited by a majority of the participants. Studies suggested that people who already inject may encourage injection initiation, enthusing about the benefits of drug injection linked to pleasure or cost-efficiency [39, 55, 56]. Encouragement by people who already inject could extend to peer pressure [57, 58], and more direct coercion [59, 60]. Other studies have also established that peers and social networks make transitioning acceptable and appealing [61, 62]. As such, the relevant authorities need to consider interventions that target social networks for prevention of transitioning. Effective peer-education interventions and those targeting social networks as part of harm reduction have been implemented elsewhere requiring little support [63–65].

In Addition, most PWIDs admitted that they still continued to initiate others into drug injection practice with increasingly regular frequency, with most of the equipment used in the injection process being sourced from friends. Sharing of needles/syringes and other injecting equipment is still ongoing among the PWIDs with over a third of all PWIDs reusing needles/syringes. This habit was much higher in Nairobi (45%) compared to the Coast region (33%) and specifically PWIDs from the Western sections of Nairobi were at an increased risk due to the high rate of sharing injecting equipment and solutions. Studies elsewhere have established that PWIDs who had shared their injection equipment at the first injection were more likely to repeat this practice over the course of their injecting career than those who had injected with new syringes (58.5% versus 16.5%; *p* = 0.003) [66, 67]. This emphasises the need for harm reduction interventions through the Needles Syringe Program (NSP) involving the provision of free syringes, needles and related injecting equipment [68].

While the overall average HIV prevalence among the PWIDs was 18.3% in the current study, most PWIDs did not think that by injecting drugs they would acquire or get infected with HIV/AIDS. HIV infection remains a major public health problem especially in Sub-Saharan Africa. This burden has been partly attributed to recreation drug use which increases the risk of HIV infection and poor adherence to ARVs [69, 70]. The risk of acquiring HIV for people who inject drugs in 2017 was 22 times higher than that for people who did not inject drugs [71]. In Kenya, 1.5 million people consisting of 18,327 injection substance users were living with HIV as at the end of 2016 [72].

## Conclusion

The current study findings indicated that a significant proportion of both male and female injecting drug users were not only at risk of acquiring and transmitting HIV through the habit of sharing drug injection equipment (Nairobi in 45%, Coast in 33%), but also through high-risk sexual behaviors. Of additional concern is the potential bridging effect, whereby an epidemic, initially fueled by the sharing of contaminated injecting equipment, is spread through sexual transmission from PWIDs to non-injecting populations and through perinatal transmission to newborns. This vulnerability underscores the need for responsive programming to better meet the specific and comprehensive needs of both male and female PWIDs.

Therefore, the most effective way to reduce the risk of contracting HIV among PWIDs who share injecting equipment is to provide free injecting equipment through the needles syringe program (NSP) as well as free condoms and lubricants. Additionally treating drug dependence as a prevention strategy can be adopted through provision of Methadone Assisted Therapy (MAT or OST) which involves ingestion of Methadone as a substitute to heroine but does not have similar addiction properties. This study therefore advocates that curbing the high HIV infection among people who inject drugs, it is necessary to implement programs in behavioral change, harm reduction through NSP and medication-assisted treatment (MAT), including opioid treatment programs (OTPs), which combines behavioral therapy and medications to treat substance use disorders.

## Data Availability

Not Applicable.

## Abbreviations

AIDS: Acquired Immuno-deficiency Virus
CBO: Community Based organization
FBO: Faith Based organization
FGD: Focus Group Discussion
HIV: Human Immunodeficiency Virus
IDU: Injecting Drug User
IQR: Inter-Quartile Range
KII: Key Informant Interview
NACADA: National Campaign against Drugs and Alcohol Abuse
NACC: National AIDS Control Council
NASCOP: National AIDS and Sexually Transmitted Diseases Programme
NGO: Non Governmental Organization
PWIDs: People Who Inject Drugs

## References

[1] UNODC (2016). World drug report 2016.

[2] Syvertsena JL, Agot K, Ohaga S, Strathdee SA, Camlind CS, Omanga E (2015). Evidence of injection drug use in Kisumu, Kenya: implications for HIV prevention. Drug Alcohol Depend..

[3] Werb D, Kerr T, Nosyk B, Strathdee S, Montaner J, Wood E (2013). The temporal relationship between drug supply indicators: an audit of international government surveillance systems. Br Med J..

[4] NACADA (2012). Rapid situation assessment of the status of drug and substance abuse National Commission against Drug Abuse, Kenya.

[5] UNODC (2019). World drug report. United Nations Office on Drugs and Crime.

[6] Ndetei D (2004). Study on the assessment of the linkages between drug abuse, injecting drug abuse and HIV/AIDS in Kenya: a rapid situation assessment.

[7] International Narcotics Control Board (2008). Report of the International Narcotics Control Board for 2008.

[8] UNODC (2007). World drug report.

[9] Renton A, Gzirishvilli D, Gotsadze G, Godinho J (2006). Epidemics of HIV and sexually transmitted infections in Central Asia: trends, drivers and priorities for control. Int J Drug Pol..

[10] Buve A, Bishikwabo-Nsarhaza K, Mutangadura G (2002). The spread and effect of HIV-1 infection in sub-Saharan Africa. Lancet..

[11] UNODC, World Drug Report 2016 (United Nations publication, Sales No. E.16.XI.7). https://www.unodc.org/doc/wdr2016/WORLD_DRUG_REPORT_2016_web.pdf.

[12] National AIDS Control Council. Kenya AIDS response Progress report 2014: Progress towards zero. Nairobi; 2014. https://www.unaids.org/sites/default/files/country/documents/KEN_narrative_report_2014.pdf

[13] Crosby GM, Stall RD, Paul JP, Barrett DC (2000). Substance use and HIV risk profile of gay/bisexual males who drop out of substance abuse treatment. AIDS Educ Prev..

[14] UNAIDS (2019). Global AIDS report 2019.

[15] Musyoki H, Bhattacharjee P, Blanchard AK, Kioko J, Kaosa S, Anthony J (2018). Changes in HIV prevention programme outcomes among key populations in Kenya: data from periodic surveys, 1–16.

[16] Volz E, Wejnert C, Degani I, Heckathorn DD (2007). Respondent-driven sampling analysis tool (RDSAT) version 5.6.

[17] Heckathorn DD (1997). Respondent-driven sampling: a new approach to the study of hidden populations. Soc Probl..

[18] Heckathorn DD (2002). Respondent-driven sampling II: deriving valid population estimates from chain-referral samples of hidden populations. Soc Probl..

[19] Erickson BH (1979). Some problems of inference from chain data. Sociol Methodol..

[20] Biernacki P, Waldorf D (1981). Snowball sampling: problems and techniques of chain referral sampling. Sociol Methods Res..

[21] Salganik MJ, Heckathorn DD (2004). Sampling and estimation in hidden populations using respondent-driven sampling. Sociol Methodol..

[22] Semaan S, Lauby J, Liebman J (2002). Street and network sampling in evaluation studies of HIV risk-reduction interventions. AIDS Rev..

[23] Goodman L (1961). Snowball sampling. Ann Math Stat..

[24] WHO questionnaire source: https://www.who.int/substance_abuse/activities/en/WHODrugInjectionStudyOperationsManual.pdf?ua=1 downloaded on 29-09-2020.

[25] Fisher AA, Laing JE, Stoeckel JE, Townsend JW. Handbook for family planning operations research. 2nd Ed, second printing ISBN 0-87834-059-9. Population Council. One Dag Hammarskjold Plaza, New York 10017; 1998.

[26] Marshall MN (1996). The key informant techniques. Fam Pract..

[27] Breen RL (2006). A practical guide to focus-group research. J Geogr High Educ..

[28] National AIDS and STI Control Programme, Ministry of Public Health and Sanitation, Kenya (2008). Guidelines for HIV Testing and Counselling in Kenya.

[29] Statistical Analyses Software (SAS) version 9.2 SAS Institute Inc., SAS 9.2 Help and Documentation, Cary, NC: SAS Institute Inc., 2002–2004.

[30] Koozegar M, Shahesmaeili A, Noroozi M (2018). Transition from first drug use to regular injection among people who inject drugs in Iran. Addict Health..

[31] Green TC, Kershaw T, Lin H, Heimer R, Goulet JL, Kraemer KL (2010). Patterns of drug use and abuse among aging adults with and without HIV: a latent class analysis of a US veteran cohort. Drug Alcohol Depend..

[32] Bluthenthal R, Chu D, Wenger L, Valente T, Kral A (2015). Does type of drug lead to quicker onset of injection?. Drug Alcohol Depend..

[33] O'Keefe D, Horyniak D, Dietze P (2016). From initiating injecting drug use to regular injecting: retrospective survival analysis of injecting progression within a sample of people who inject drugs regularly. Drug Alcohol Depend..

[34] Budambula V, Matoka C, Ouma J, Ahmed AA, Otieno MF, Were T (2018). Socio-demographic and sexual practices associated with HIV infection in Kenyan injection and non-injection drug users. BMC Public Health..

[35] Budambula V, Budambula LMN (2017). Chasing the dragon: drug use and abuse ISBN-10: 9966103023/ISBN-13: 978–9966103024.

[36] Atkinson J, McCurdy S, Williams M, Mbwambo J, Kilonzo G (2011). HIV risk behaviors, perceived severity of drug use problems, and prior treatment experience in a sample of young heroin injectors in Dar es salaam, Tanzania. African J Drug Alcohol Stud.

[37] Odinokova V, Rusakova M, Urada LA, Silverman JG, Raj A (2014). Police sexual coercion and its association with risky sex work and substance use behaviours among female sex workers in St Petersburg and Orenburg, Russia. Int J Drug Policy.

[38] Baluku M, Wamala T, Muhangi D (2019). HIV- and hepatitis C-related risk behaviors among people who inject drugs in Uganda: implications for policy and programming. Harm Reduction J..

[39] Amin-Esmaeili M, Rahimi-Movaghar A, Gholamrezaei M, Razaghi E (2016). Profile of people who inject drugs in Tehran, Iran. Acta Med Iran.

[40] Tuchman E (2015). Women’s injection drug practices in their own words: a qualitative study. Harm Reduct J..

[41] Spittal, P. M., Craib, K. J. P., Wood, E., Laliberté, N., Li, K., Tyndall, M. W., O’Shaughnessy. Female injection drug users in Vancouver. CMAJ. 166(7):894–9. PMC100922 11949985

[42] Des Jarlais DC, Feelemyer JP, Modi SN, Arasteh K, Hagan H (2012). Are females who inject drugs at higher risk for HIV infection than males who inject drugs: an international systematic review of high seroprevalence areas. Drug Alcohol Depend..

[43] Gajendra KM, Jagadish M, Ramesh SP, Rajatashuvra A, Senjam GS, Akoijam SB (2012). Factors associated with ever HIV testing among injecting drug users (IDUs) in two HIV high prevalent states of India. Indian J Med Res..

[44] Medhi KG, Jagadish M, Ramesh SP, Rajatashuvra A, Senjam GS (2012). Factors associated with ever HIV testing among injecting drug users (IDUs) in two HIV high prevalent states of India. Indian J Med Res..

[45] Swe LA, Nyo KK, Rashid A (2010). Risk behaviours among HIV positive injecting drug users in Myanmar: a case control study. Harm Reduction J..

[46] Khajedaluee M, Dadgarmoghaddam M, Erfanian M, Alipourtabrizi A, Khadem-Rezaiyan M (2015). Women, drug dependency and consequences: a study from a developing country. J Addict..

[47] Mehrabi M, Eskandarieh S, Khodadost M, Sadeghi M, Nikfarjam A, Hajebi A (2016). The impact of social structures on deviant behaviors: the study of 402 high risk street drug users in Iran. J Addict..

[48] Mars SG, Ondocsin J, Ciccarone D (2018). Toots, tastes and tester shots: user accounts of drug sampling methods for gauging heroin potency. Harm Reduct J..

[49] Ciccarone D, Unick GJ, Cohen JK, Mars SG, Rosenblum D (2016). Nationwide increase in hospitalizations for heroin-related soft tissue infections: associations with structural market conditions. Drug Alcohol Depend..

[50] Barry D, Syed H, Smyth BP (2012). The journey into injecting heroin use. Heroin Addict Related. Clin Probl.

[51] Trenz RC, Scherer M, Harrell P, Zur J, Sinha A, Latimer W (2012). Early onset of drug and polysubstance use as predictors of injection drug use among adult drug users. Addict Behav..

[52] Ellen T (2015). Women’s injection drug practices in their own words: a qualitative study. Harm Reduction J..

[53] Li J, Liu H, Li J, Luo J, Jarlais DD, Koram N (2011). Role of sexual transmission of HIV among young non-injection and injection opiate users: a respondent driven sampling study. Sex Transm Dis..

[54] Noor SWB, Ross MW, Lai D, Risser JM (2014). Drug and sexual HIV risk behaviours related to knowledge of HIV serostatus among injection drug users in Houston, Texas. Int J STD AIDS.

[55] Guise A, Dimova M, Ndimbii J, Clark P, Rhodes T (2015). A qualitative analysis of transitions to heroin injection in Kenya: implications for HIV prevention and harm reduction. Harm Reduct J..

[56] Kolla G, Strike C, Roy E, Altenberg J, Balian R, Silver R (2015). Initiation stories: an examination of the narratives of people who assist with a first injection. Subst Use Misuse..

[57] Rhodes T, Bivol S (2012). Back then and nowadays: social transition narratives in accounts of injecting drug use in an east European setting. Soc Sci Med..

[58] Robertson AM, Lozada R, Pollini RA, Rangel G, Ojeda VD (2012). Correlates and contexts of US injection drug initiation among undocumented Mexican migrant men who were deported from the United States. AIDS Behav..

[59] Briggs D, Rhodes T, Marks D, Kimber J, Holloway G, Jones S (2009). Injecting drug use and unstable housing: scope for structural interventions in harm reduction. Drugs-Educ Prev Policy..

[60] Lankenau SE, Wagner KD, Bloom JJ, Sanders B, Hathazi D, Shin C (2010). The first injection event: differences among heroin, methamphetamine, cocaine, and ketamine initiates. J Drug Issues..

[61] Harocopos A, Goldsamt LA, Kobrak P, Jost JJ, Clatts MC (2009). New injectors and the social context of injection initiation. Int J Drug Policy..

[62] Kermode M, Longleng V, Singh B, Bowen K, Rintoul A (2009). Killing time with enjoyment: a qualitative study of initiation into injecting drug use in north-East India. Subst Use Misuse..

[63] Degenhardt L, Bradley M, Vickerman P, Rhodes T, Latkin C, Hickman M (2010). Prevention of HIV infection for people who inject drugs: why individual, structural, and combination approaches are needed. Lancet..

[64] Weeks MR, Dickson-Gomez J, Mosack KE, Convey M, Martinez M, Clair S (2006). The risk avoidance partnership: training active drug users as peer health advocates. J Drug Issues..

[65] Weeks MR, Li J, Dickson-Gomez J, Convey M, Martinez M, Radda K (2009). Outcomes of a peer HIV prevention program with injection drug and crack users: the risk avoidance partnership. Subst Use Misuse..

[66] Oliveira MLA, Hacker MA, Oliveira SA, Telles PR, Bastos FI (2006). The first shot: the context of first injection of illicit drugs, ongoing injecting practices, and hepatitis C infection in Rio de Janeiro, Brazil. Cadernos de Saúde Pública.

[67] Novelli LA, Sherman SG, Havens JR, Strathdee SA, Sapun M (2005). Circumstances surrounding the first injection experience and their association with future syringe sharing behaviors in young urban injection drug users. Drug Alcohol Depend..

[68] Needle R, Zhao L (2010). HIV prevention among injection drug users: closing the coverage GAP, expanding access, and scaling up Core interventions.

[69] Wakibi SN, Ng’ang’a ZW, Mbugua GG (2011). Factors associated with non-adherence to highly active antiretroviral therapy in Nairobi, Kenya. AIDS Res Ther.

[70] Goodman J, Packard MG (2016). Memory systems and the addicted brain. Front Psychiatry..

[71] UNAIDS (2018). Global AIDS report 2018. Miles to go: closing gaps, breaking barriers, righting injustices. Geneve United Nations Programme on HIV/AIDS.

[72] Kenya PR, Oguya F, Ongecha FA (2011). Assessment of HIV prevalence and related behaviour among IDUs in Kenya: UNODC.

